# The Standard of Mental Administration

**Published:** 1915-05-01

**Authors:** 


### THE STANDARD OF MENTAL ADMINISTRATION.

### Sir Thomas Clouston's Theories and Practice.

Those whose habit it is to praise the past would find little comfort in the consideration of the methods made use of not so very many years ago in dealing with the mentally afflicted. It was not till towards the end of the eighteenth century that Pinel—despite the warnings of less courageous and less humane critics—had the chains removed from the patients under his care; and about the same time Tuke was commencing at York his beneficent work on behalf of the insane. The good seed which they had sown came but slowly to fruition. The "Report on Madhouses," published in 1815, revealed an appalling state of affairs even in London: chains and scourgings and filth indescribable. It was not until the third and fourth decades of the nineteenth century that the efforts of such men as Hill and Conolly made it clear that mechanical restraints and coercions were unscientific, inhumane, and unnecessary. The period of enlightenment may be dated—for all practical purposes—from this epoch. The possibility of reforms as comprehensive as these no longer existed. Yet much remained to be done in the application of these more humane methods throughout the country; and beyond that in the general amelioration of the conditions in which the insane lived. More than this—there was the necessity of instructing their potential guardians that they were not to regard themselves as the keepers of furious wild beasts or the flagellators of fiend-ridden human outcasts, but as nurses of sick people whose nervous mechanism was disordered. Among those who helped largely in diffusing the new knowledge in regard to insanity during the latter half of the nineteenth century, the late Sir Thomas Clouston undoubtedly occupies a prominent position. He was in various ways admirably qualified so to do. To an unusual power of administrative ability, which was exhibited in the carrying out of reforms in the nursing of the insane and in the structural alterations which he initiated or supervised, there was united the capacity for the accurate observation of clinical symptoms and for the lucid exposition of the knowledge he had acquired.

His methods of treatment were not founded on a blind empiricism. He had a rational and secure basis for the faith that was in him. Mental disorder, he believed, is dependent on brain derangement; and it is, therefore, necessary to deal with this physical substratum and to endeavour to bring it back, if possible, to orderly functioning. In his own words as they appear in one of his later books, "The Hygiene of Mind," "mental hygiene must be based upon the assumption that mind, in its every faculty and exhibition, implies and requires accompanying brain action . . . 'No brain, no mind' must be an unquestioned axiom to the student of Mental Hygiene." That being so, it follows that everything must be done to make the stimuli to which the body is subjected as pleasurable and health-giving as possible. Monotony must be avoided. Hence arose his desire to modify the environment for his patients so that variety of visual impressions should, where necessary, obviate the tedium which must inevitably result from an ever-recurring repetition. In such an apparently trivial matter as that of the furnishings and decorations of rooms he realised the scope there was for the introduction of a pleasing diversity. "Asylums for the insane have become pleasant hospitals and homes, largely through the slow development of suitable occupations and amusements for the patients," he remarks in that monument of clinical record the "Lectures on Mental Diseases." Another aspect of treatment on which he laid great stress was that of feeding. He preached the gospel of fatness—the need for improving the nutrition of the patient by every possible means. "Fatten and nourish your patients is," he says "a rule to which there are marvellously few exceptions in mental medicine." In regard to drug treatment, much of what he wrote was based upon original investigation; and this was especially so in the matter of narcotics and sedatives.

Of his published work, that which deals with the "Neuroses of Development" has almost attained to classic importance. This is not the place to enter upon the vexed question of adolescent insanity *versus* dementia praecox; but even the most ardent disciple of Kraepelin will admit the sterling value of Clouston's researches into the developmental insanities and the wisdom of his plea for the more rational, more physiological methods of education. In his other writings he has spread abroad the knowledge of mental disorders; and his powers of vivid exposition have made certain of his books acceptable to a much wider circle than would have been the case if his literary gifts had been of a more circumscribed nature. As a lecturer he exhibited the same facility of expression. His lectures were no dry-as-dust disquisitions through which he mechanically plodded; they were eloquent discourses interspersed with anecdotes and references to characters in literature which served to illustrate the subject under discussion. His clinical demonstrations were equally admirable; in an arresting manner he called attention to the prominent morbid mental symptoms, discussed prognosis and treatment, and left an impression upon his hearers which time does not efface.

His death has removed from the ranks of psychiatrists one who had done very much to raise the standard of efficiency in administrative work; a lucid exponent of extensive knowledge; an accomplished literary man; and, not least, a teacher who not only stimulated his students to an enthusiastic appreciation of the subject he had so much at heart, but who at the same time attracted their esteem and affection. So far back as October 22, 1892, The Hospital contained a detailed account of Clouston's work, which still remains a striking record of the progress that he effected.

